# Comparison of insulin resistance indices in predicting albuminuria among patients with type 2 diabetes

**DOI:** 10.1186/s40001-023-01134-2

**Published:** 2023-05-10

**Authors:** Seyed Ali Nabipoorashrafi, Azam Adeli, Seyed Arsalan Seyedi, Soghra Rabizadeh, Razman Arabzadeh Bahri, Fatemeh Mohammadi, Amirhossein Yadegar, Manouchehr Nakhjavani, Alireza Esteghamati

**Affiliations:** Endocrinology and Metabolism Research Center (EMRC), School of Medicine, Vali-Asr Hospital, P.O.Box 13145784, Tehran, Iran

**Keywords:** Chronic kidney disease, Lipid accumulation product index, Triglyceride-glucose index, Visceral adiposity index, Diabetes mellitus

## Abstract

**Purpose:**

Diabetes is the leading cause of kidney disease. Up to 40% of the population with diabetes experience diabetic kidney disease (DKD). The correlation of DKD with insulin resistance (IR) indices has been shown in previous studies. In this study, the objective was to evaluate surrogate IR indices, including the Triglyceride-Glucose (TyG) index, Visceral Adiposity Index (VAI), Lipid Accumulation Product (LAP), and Homeostasis Model Assessment of Insulin Resistance (HOMA-IR) to find the most valuable index for the correlation between albuminuria and IR in the type 2 diabetes (T2D) population. Albuminuria is defined as urine albumin excretion of > 30 mg/day.

**Methods:**

In this cross-sectional study, 2934 participants were enrolled and evaluated for urinary albumin excretion, and albuminuria was detected in 526 of the entries. The logistic regression models and Receiver Operating Characteristic (ROC) curve analysis were performed to assess the relationship of TyG index, VAI, LAP, and HOMA-IR's with albuminuria in patients with T2D.

**Results:**

The TyG index had the highest association (OR 1.67) with the presence of albuminuria in patients with T2D, followed by HOMA-IR (OR 1.127), VAI (OR 1.028), and LAP (OR 1.004). These four indices remained independent after adjustment for multiple confounders. Based on the ROC curve, TyG revealed the best area under the curve (AUC) for revealing albuminuria with sufficient accuracy (AUC: 0.62) in comparison with other measured indices. The calculated TyG index cut-off point for the presence of albuminuria was 9.39.

**Conclusion:**

Among the indices, TyG index had the most significant correlation with albuminuria in patients with T2D.

## Introduction

Diabetes is the most common metabolic disorder with numerous micro- and macrovascular complications, including, diabetic nephropathy [1, 2]. Albuminuria, defined as the presence of albumin in the urine (albumin excretion rate > 30 mg/d), has been highly correlated to kidney damage and is detectable in the early stages of Chronic Kidney Disease (CKD) [3]. CKD is a global health issue associated with a high rate of complications. It is approximated that more than 850 million people suffer from kidney diseases around the world, the majority of whom showed evidence of CKD [4]. Although several pathogenic mechanisms are associated with the onset and progression of CKD, diabetes mellitus is the leading cause of the disease, and approximately 40% of patients with T2D experience diabetic kidney disease [5, 6]. Nearly half of patients (51%) who receive dialysis therapy suffer from diabetes mellitus as the primary origin of their kidney failure [7, 8].

Studies have indicated that IR and related mechanisms are pathogenic factors for renal failure [9]. IR is a major pathogenic mechanism in T2D but also can be found in patients with Type 1 Diabetes (T1D) [10]. IR is detectable in the early stages of CKD. In addition, as patients' renal function deteriorates, the IR level increases [11].

Although the gold standard method for evaluation of IR is hyperinsulinemic-euglycemic clamp (insulin clamp), this technique is costly and rather sophisticated to perform in clinic [12, 13]. Another indicator used for the assessment of the IR level is the HOMA-IR [14]. However, in the case of patients with T2D, using the HOMA-IR method for the measurement of IR is not reliable enough since a considerable proportion of patients with T2D receive insulin therapy [15, 16].

Therefore, several attempts have been made to discover other indices that could be more accessible for assessing IR [17]. Furthermore, in addition to HOMA-IR, the TyG, VAI, and LAP index have been identified as the surrogate indices for IR [18–20]. Several studies have shown an association between these indices and IR levels [21–25].

To the best of our knowledge, there has been no relevant literature that compares the relationship between these four indices and albuminuria in patients with T2D. Thus, the present study was aimed to evaluate this relationship and to identify the cut-off point for the most valuable index to reveal albuminuria in patients with T2D.

## Materials and methods

This is a cross-sectional study of an ongoing cohort which was carried out in the outpatients Diabetes Clinic of Vali-Asr Hospital (affiliated with Tehran University of Medical Sciences), a large tertiary referral center. All eligible patients with T2D who attended the outpatient clinic between 2011 and 2021 were enrolled consecutively in the study.

The ethical committee of the Tehran University of Medical Sciences (TUMS) approved the study with the registered number of IR.TUMS.IKHC.REC.1400.085.

T2D was diagnosed according to the 2022 American Diabetes Association guidelines [26], and albuminuria was characterized by persistent albuminuria (> 30 mg/d) that was confirmed on two routine check-ups 3–6 months apart.

Applied exclusion criteria were non-diabetic renal diseases, feverish infection or history of infectious disease in the last six months, any chronic disease (e.g., cardiac failure, liver failure, malignancy, any kind of insulin therapy, patients with T1D, other special types of diabetes, benign prostatic hyperplasia, severe uncontrolled hypertension, severe hyperglycemia), and recent excessive exercise.

Demographic factors and clinical data of participants, including age, sex, height, weight, waist circumference, blood pressure (systolic and diastolic), duration of diabetes, and medications for diabetes and dyslipidemia were recorded.

The body weight and height of enrolled patients were measured to the nearest 0.1 kg and 0.001 m, respectively. Waist circumference was calculated at the central point between the lower level of the lowest palpable rib and the highest of the iliac crest to the nearest 0.001 m. Blood pressure was evaluated after a ten-minute rest. The measurement was repeated after 15 min, and the average was recorded as the blood pressure value.

Blood samples were taken after ten or more hours of fasting at 8:00 am. Fasting blood sugar (using hexokinase enzyme method) and fasting insulin (using radioimmunoassay techniques (Immunotech, Prague, Czech Republic)) were measured.

Participants' serum creatinine level was evaluated by the Jaffe technique (Pars Azmun, Karaj, Iran). Accuracy of random samples were evaluated by the central reference laboratory (Tehran, Iran), which the results and kits approval was met. Hemoglobin A1c (HbA1c) was measured by high performance liquid chromatography (A1C, DS5 Pink kit; Drew, Marseille, France). Fasting plasma glucose (FPG) and 2-h postprandial (2hPP) glucose measurements were conducted using enzymatic colorimetric methods by the glucose oxidase test. The estimated glomerular filtration rate (eGFR) was calculated by the Chronic Kidney Disease Epidemiology Collaboration Equation [27]. HOMA-IR, a reliable surrogate of peripheral insulin resistance, was then calculated as FPG (mg/dL) multiplied by insulin (IU), divided by 405. Serum lipids concentration including total cholesterols, triglycerides (TG), high-density lipoprotein cholesterol (HDLC), and low-density lipoprotein cholesterol (LDL-C), were measured using enzymatic methods (Parsazmun, Karaj, Iran). In TG below 400 mg/dL, the Friedewald formula was applied for the calculation of Plasma Low-Density Lipoprotein Cholesterol (LDL-C) (mg/dL) [28]. This formula is an estimation of LDL-C level based on TC, TG, and HDL-C levels as described below:

$${\text{LDL-}}{\text{C }}\left( {{\text{mg}}/{\text{dL}}} \right)\, = \,{\text{TC }}\left( {{\text{mg}}/{\text{dL}}} \right)\, - \,{\text{HDL-}}{\text{C }}\left( {{\text{mg}}/{\text{dL}}} \right)\, - \,{\text{TG }}\left( {{\text{mg}}/{\text{dL}}} \right)/{5}$$

Otherwise, the enzymatic method was used. Also, two hours later (10:00 am), 2-h postprandial glucose (2 h-PPG) was measured through the hexokinase enzyme method.

The Estimated glomerular filtration rate (eGFR) was calculated in participants by the Cockcroft-Gault formula [29] as described below.

Creatinine clearance (ml/min): [(140 − age) × body weight] /[plasma creatinine × 72] (× 0.85 if female).

The definition of TyG, VAI, LAP, and HOMA-IR indices are:

The TyG index was defined as the Ln [fasting TG (mg/dL) * fasting glucose (mg/dL)/2] for both genders.

The VAI index was calculated through sex-specific formulas; males (waist circumference (cm)/(39.68 + (1.88*BMI)) *(TG/1.03) *(1.31/HDL-C); females: (waist circumference (cm)/(36.58 + (1.89*BMI)) * (TG/0.81) *(1.52/HDL-C), where both TG and HDL-C levels are reported in mmol/L.

The LAP index was determined as (waist circumference(cm) – 65) * (TG (mmol/L)) in males and (waist circumference(cm) − 58) * (TG (mmol/L)) in females.

The HOMA-IR index was calculated by: fasting glucose in mmol/l*fasting insulin in μU/ml/22.5

### Statistical analysis

SPSS version 24 was used for statistical analysis. The normality of the data was evaluated using Kolmogorov–Smirnov tests. Continuous variables with normal distribution are provided as mean ± standard deviation and categorical variables are presented as frequency and percentage (%).

The TyG, HOMA-IR, LAP, and VAI indices, were compared among patients with and without albuminuria by univariate analyses, using t-tests to compare continuous variables and chi-square for categorical ones between the groups.

ROC curve analysis was performed and the Youden index was used to calculate the optimized cut-offs value of TyG, HOMA-IR, LAP, and VAI indices.

The Logistic regression models were used to measure the relationship between the indices and albuminuria among T2D patients in univariate and multivariate logistic regression analyses. A univariate logistic regression analysis was conducted on each index. Two multivariable models were constructed for each index. Model 1 was adjusted for sex, age, blood pressure; other models (model 2) were adjusted for the duration of diabetes, blood LDL-C level, retinopathy, lipid-lowering agents use (divided into three categories), and waist circumference (was not adjusted in models which are included the LAP and VAI indices due to their formulas) in addition to model 1's confounders. Finally, model 2 was adjusted for HOMA-IR to demonstrate whether these indices remained independent (model 3). P-value less than 0.05 was considered statistically significant during analysis.

## Results

A total of 2934 participants with T2D were included in this study. Patients were divided into two groups depending on the presence of albuminuria. In 526 participants albuminuria was detected, and 2408 T2D patients did not meet the albuminuria criteria.

The mean age of participants was 56.46 years, of whom 52.9% were women. Albuminuria was more common in men (52.9%). In addition, 54.2% of patients without albuminuria were women.

Those with albuminuria had higher diastolic blood pressure and longer duration of diabetes (P- value < 0.001).

Also, more proportion of patients with albuminuria (140 patients: 26.7%) had cardiovascular diseases compared to another group (530 patients: 22.0%). Similarly, the proportion of patients with retinopathy in the group with albuminuria (128 patients: 24.4%) was higher than the other (222 patients: 9.2%). In addition, patients with albuminuria had higher FBS (182.39 mg/dL), HbA1c (8.32%), and Triglyceride (207.34 mg/dL) levels.

The total cholesterol and LDL-C were higher in patients with T2D and albuminuria compared to those without albuminuria (192.23 ± 50.97 VS. 179.38 ± 44.59, and 111.64 ± 69.36 VS. 101.56 ± 34.36 mg/dL, respectively (both P-values < 0.001)).

Moreover, TyG, HOMA-IR, LAP, and VAI indices were higher in patients with albuminuria (all P-values < 0.001).

However, age, non-HDL-C, HDL-C, eGFR, and the HbA1c did not show a significant difference (all P-values > 0.05) between the two groups (Table 1).

**Table 1:** Demographic and metabolic characteristics of participants stratified by albuminuria

Characteristics	with Albuminuria	without Albuminuria	P.value
	N = 526	N = 2408	
Age (year)	56.50 ± 9.68	56.28 ± 10.83	0.68
Female n (%)	249 (47.1%)	1305 (54.2%)	< 0.004
BMI^a^	30.01 ± 4.86	29.54 ± 4.76	0.041
BP^b^ Systolic (mmHg)	134.67 ± 18.62	137.50 ± 49.71	0.075
BP^b^ Diastolic (mmHg)	81.62 ± 9.17	79.35 ± 9.23	< 0.001
FBS (mg/dl)^c^	182.39 ± 60.34	160.83 ± 58.70	< 0.001
2hpp (mg/dl)^d^	263.86 ± 93.98	218.77 ± 82.88	< 0.001
HbA1c (%)^e^	8.32 ± 1.67	7.64 ± 1.54	< 0.001
Cholesterol (mg/dl)	192.23 ± 50.97	179.38 ± 44.59	< 0.001
HDL-C (mg/dl)^f^	44.41 ± 12.310	45.08 ± 11.096	0.49
LDL-C (mg/dl)^g^	111.64 ± 69.36	101.5 ± 34.36	< 0.001
TG (mg/dl)^h^	207.34 ± 137.70	175.17 ± 99.60	< 0.001
non-HDL-C (mg/dl)	148.43 ± 49.84	143.61 ± 42.11	0.074
eGFR (ml/min)^i^	93.75 ± 29.28	91.59 ± 25.22	0.118
Waist circumference (cm)	101.52 ± 9.89	99.29 ± 9.60	< 0.001
Cardiovascular disease (%)	140 (26.7%)	530 (22.0%)	< 0.02
Hypertension (%)	285 (54.2%)	1009 (41.9%)	< 0.001
Retinopathy (%)	128 (24.4%)	222 (9.2%)	< 0.001
Duration of diabetes (years)	11.54 ± 7.69	9.54 ± 7.00	< 0.001
TyG^j^	9.64 ± 0.69	9.37 ± 0.63	< 0.001
LAP^k^	94.54 ± 72.90	76.05 ± 49.13	< 0.001
VAI^l^	8.50 ± 7.50	7.13 ± 5.10	< 0.001
HOMA-IR^m^	4.55 ± 2.72	3.79 ± 2.32	< 0.001
Lipid-lowering agents (%)			
NO medication (%)	204 (38.8%)	685 (28.5%)	
User of Fibrate (%)	20 (3.8%)	75 (3.1%)	< 0.001
User of Statin (%)	302 (57.4%)	1648 (68.4%)	
Antihypertensive agents (%)	285 (54.2%)	1009 (41.9%)	< 0.001

^a^Body mass index, ^b^Blood pressure, ^c^Fasting blood sugar, ^d^2-hour postprandial glucose, ^e^Hemoglobin A1c, ^f^High-density lipoprotein, ^g^Low-density lipoprotein, ^h^Triglycerides, ^i^Estimated glomerular filtration rate, ^j^Triglyceride-glucose, ^k^Lipid Accumulation Product, ^I^Visceral Adiposity Index, ^m^Homeostasis Model Assessment of Insulin Resistance

The multivariable logistic regression analysis revealed that TyG, HOMA-IR, LAP, and VAI were positively correlated with the incidence of albuminuria among patients with T2D, and they remained independent after adjustments for multiple confounders in all models. Furthermore, these models showed that the TyG index and albuminuria had the strongest association (OR 1.674 95% CI (1.376–2.037; P-value < 0.001), followed by HOMA-IR (OR 1.127; 95% CI 1.072–1.185; P-value < 0.001), VAI (OR 1.028; 95% CI 1.009–1.047; P-value < 0.003) and LAP (OR 1.004; 95% CI 1.002–1.006; P-value < 0.001). The regression analysis demonstrated that with each unit increase of the TyG index, the risk of developing albuminuria increased by 1.67 times, and this relation was independent of HOMA-IR (Table 2).

**Table 2:** Logistic regression analysis for the presence of Albuminuria in T2D

Indices	Model 0			Model 1			Model 2			Model 3		
	R squared	OR (95% CI)	P-value	R squared	OR (95% CI)	P-value	R squared	OR (95% CI)	P-value	R squared	OR (95% CI)	P-value
TyG^a^	0.040	1.859(1.608–2.148)	< 0.001	0.069	1.77 (1.53–2.06)	< 0.001	0.116	1.72 (1.44–2.04)	< 0.001	0.132	1.674 (1.37–2.03)	< 0.001
VAI^b^	0.013	1.038(1.022–1.053)	< 0.001	0.052	1.041 (1.02–1.05)	< 0.001	0.100	1.037 (1.01–1.05)*	< 0.001	0.119	1.028 (1.009–1.047) *	< 0.003
LAP^c^	0.024	1.005(1.004–1.007)	< 0.001	0.060	1.005(1.004–1.007)	< 0.001	0.101	1.005(1.003–1.007)*	< 0.001	0.121	1.004(1.002–1.006) *	< 0.001
HOMA-IR^d^	0.022	1.124(1.080–1.169)	< 0.001	0.067	1.11(1.07–1.16)	< 0.001	0.114	1.13 (1.07–1.18)	< 0.001	–	–	–

Model 0 is a univariate logistic regression with no adjustment. Model 1 was adjusted for age, sex, duration of diabetes, systolic and diastolic blood pressure. Model 2 was adjusted for age, sex, duration of diabetes, systolic and diastolic blood pressure, waist circumference, retinopathy, lipid-lowering agents, and LDL-C. Model 2 was adjusted for HOMA-IR in Model 3

^*^Waist circumference was not adjusted in these models due to their formulas

^a^Triglyceride-glucose index, ^b^Visceral Adiposity Index, ^c^Lipid Accumulation Product, ^d^Homeostasis Model Assessment of Insulin Resistance

**Fig. 1:**
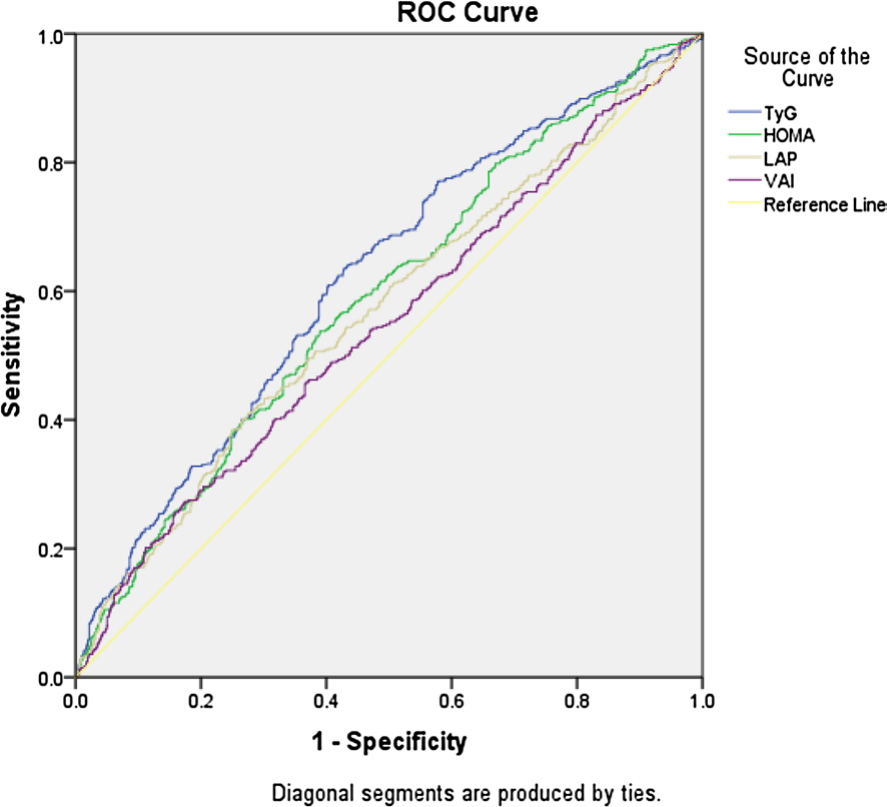
ROC curves for TyG, HOMA-IR, LAP and VAI for revealing albuminuria

The ROC curve was utilized to evaluate the significance of these indices as an indicator for albuminuria, and it was determined that the TyG index plot had the largest AUC with sufficient accuracy (0.62 (range, 0.59–0.65)). On the other hand, the results showed that VAI had the smallest AUC 0.55 (range, 0.52–0.58), and the calculated AUC for HOMA-IR and LAP were 0.59 (range, 0.57–0.62) and 0.57 (range, 0.54–0.60), respectively (Fig. 1).

The best-calculated TyG cut-off for albuminuria evaluation was 9.39 with 0.64 and 0.55 sensitivity and specificity, respectively (Table 3).

**Table 3:** AUC, sensitivity and specificity by the optimized cutoff points for TyG, HOMA-IR, LAP, and

Indices	Cut-off	Sensitivity%	Specificity%	^e^ AUC (95% CI)	P-value
TyG^a^	9.39	64	55	0.621 (0.593–649)	< 0.001
HOMA-IR^b^	3.81	54	61	0.590 (0.561–0.618)	< 0.001
LAP^c^	93.19	39	75	0.570 (0.540–0.600)	< 0.001
VAI^d^	6.90	48	61	0.547 (0.517–0.577)	< 0.001

^a^Triglycerides-glucose, ^b^Homeostasis Model Assessment Insulin Resistance, ^c^Lipid Accumulation Product, ^d^Visceral Adiposity Index, ^e^Area Under the ROC Curve

## Discussion

This study evaluated the potential association between TyG, LAP, VAI, HOMA-IR indices, and albuminuria in patients with T2D. This study’s main aim was to investigate which index was the most valuable for revealing albuminuria in T2D patients. In this cross-sectional study, we found that TyG, LAP, VAI, and HOMA-IR indices all had strong positive associations with albuminuria. This relationship remained significant after adjustments for multiple confounders. In addition, all of these models indicated that the TyG index was the best indicator of albuminuria independent of HOMA-IR. Also, the TyG index showed the greatest AUC in comparison with other indices.

There is a paucity of evidence in the literature regarding these indices and their relationship with albuminuria in patients with type 2 diabetes. Recent studies have shown that IR level is correlated with nephropathy and declining renal function [30, 31]. Due to lack of a low-cost and available technique for estimation of insulin sensitivity, TyG, LAP, VAI, and HOMA-IR indices were introduced as high-correlated indices with IR levels. Various studies have evaluated the relation of these indices with IR-dependent diseases, including diabetes, metabolic syndrome, cardiovascular diseases, and microvascular complications [32–34].

HOMA-IR is a model which has been used widely in studies and clinical practice to estimate IR [35]. VAI is a measurable index for estimating visceral adipose distribution, which is associated with vascular diseases [19, 36]. LAP is an index to assess central lipid distribution and lipotoxicity [37, 38]. LAP and VAI consist of anthropometric measures (such as waist circumference) and plasma laboratory data (such as triglyceride or HDL-C) in their formula. TyG index is another marker that has been proposed as a reliable index for evaluating the IR level, and studies emphasized its ability to evaluate microvascular damage [39].

Tang Chen et al. [40] found that the TyG index is more valuable than VAI and LAP for predicting CKD in the general population. Moreover, the mentioned study on the general Chinese population showed that LAP and VAI were not independent of albuminuria; however, in our study these associations were significant in all models.

Additionally, Dai D. et al. evaluated the relation of VAI and LAP and chronic kidney disease. They concluded LAP and VAI were related to kidney disease in a randomized rural population in China [41]. Nonetheless, in the present study, which only included patients with T2D, VAI and LAP were related to albuminuria.

Zhao S. et al. demonstrated an association between the TyG index and albuminuria among the elderly population (more than 65 years old) [42]. In parallel, our study revealed that the TyG index is associated with albuminuria among patients with T2D.

After examining LAP, VAI, and TyG indices, Fiorentino et al. concluded that the LAP index had the most correlation with IR and subclinical vascular damages. In Fiorentino’s study, subjects had been chosen from a population with different degrees of insulin sensitivity, including normal glucose tolerance, impaired glucose tolerance, impaired fasting glucose, and patients with T2D [43]. However, in this study, we evaluated patients with diabetes and IR, particularly.

Furthermore, Ying Pan et al. showed that there was a significant association between TyG index and albuminuria in hospitalized patients [44]. However, in our study we evaluated this relationship in an outpatient setting.

## Conclusion

Of the calculated indices of insulin resistance in this study, we concluded that the TyG index had the most significant correlation with albuminuria in patients with T2D. Due to the simplicity and practicality of calculating the TyG index, its utilization would be a favorable method for evaluating albuminuria. Nevertheless, extensive and longitudinal research is needed to confirm these relationships.

### Study strengths and limitations

This study compared the association of TyG, LAP, VAI, and HOMA-IR indices and albuminuria in the T2D population for the first time and indicated that all mentioned indices have a significant relationship with the presence of albuminuria. In addition, our study was conducted on a large cohort of individuals with T2D who were enrolled over a decade.

However, some shortcomings need to be interpreted in this study. First, this was a cross-sectional study that could not determine causality. Another drawback of this study was the inability to compare these indices with the hyperinsulinemic-euglycemic clamp test which is the gold standard method for detecting insulin sensitivity and glucose utilization.

## Data Availability

The dataset used in this study is available upon request from the corresponding author.
